# *Plasmodium falciparum kelch* *13* Mutations, 9 Countries in Africa, 2014–2018

**DOI:** 10.3201/eid2707.203230

**Published:** 2021-07

**Authors:** Sarah E. Schmedes, Dhruviben Patel, Simran Dhal, Julia Kelley, Samaly S. Svigel, Pedro Rafael Dimbu, Adicatou-Laï Adeothy, Gauthier Mesia Kahunu, Papy Mandoko Nkoli, Abdoul Habib Beavogui, Simon Kariuki, Don P. Mathanga, Ousmane Koita, Deus Ishengoma, Ally Mohamad, Moonga Hawela, Leah F. Moriarty, Aaron M. Samuels, Julie Gutman, Mateusz M. Plucinski, Venkatachalam Udhayakumar, Zhiyong Zhou, Naomi W. Lucchi, Meera Venkatesan, Eric S. Halsey, Eldin Talundzic

**Affiliations:** Association of Public Health Laboratories, Silver Spring, Maryland, USA (S.E. Schmedes);; Centers for Disease Control and Prevention, Atlanta, Georgia, USA (S.E. Schmedes, D. Patel, S.S. Svigel, A.M. Samuels, J. Gutman, M.M. Plucinski, V. Udhayakumar, Z. Zhou, N.W. Lucchi, E. Talundzic);; Williams Consulting LLC, Baltimore, Maryland, USA (D. Patel);; The Wallace H. Coulter Department of Biomedical Engineering, Georgia Institute of Technology, Atlanta (S. Dahl);; CDC Foundation, Atlanta (J. Kelley);; National Malaria Control Program, Ministry of Health, Luanda, Angola (P.R. Dimbu);; National Malaria Control Program, Ministry of Health, Porto-Novo, Benin (A.-L. Adeothy);; University of Kinshasa, Kinshasa, Democratic Republic of the Congo (G.M. Kahunu);; National Institute of Biomedical Research, Kinshasa (P.M. Nkoli);; Maferinyah Rural Health Research Center, Mafèrinyah, Guinea (A.H. Beavogui);; Kenya Medical Research Institute, Centre for Global Health Research, Kisumu, Kenya (S. Kariuki);; University of Malawi College of Medicine, Blantyre, Malawi (D.P. Mathanga);; University of Sciences, Techniques, and Technologies of Bamako, Bamako, Mali (O. Koita);; Harvard T.H. Chan School of Public Health, Boston, Massachusetts, USA (D. Ishengoma);; National Institute for Medical Research, Tanga Research Centre, Dar es Salaam, Tanzania (D. Ishengoma, A. Mohamad);; Monash University Faculty of Pharmaceutical Sciences, Melbourne, Australia (D. Ishengoma);; National Malaria Elimination Centre, Lusaka, Zambia (M. Hawela);; US President’s Malaria Initiative, Centers for Disease Control and Prevention, Atlanta (L.F. Moriarty, M.M. Plucinski, E.S. Halsey);; US President’s Malaria Initiative, US Agency for International Development, Washington, DC, USA (M. Venkatesan)

**Keywords:** *Pfk13* mutations, *Plasmodium falciparum*, *kelch 13*, artemisinin resistance, molecular surveillance, Africa, malaria, parasites, antimicrobial resistance

## Abstract

The spread of drug resistance to antimalarial treatments poses a serious public health risk globally. To combat this risk, molecular surveillance of drug resistance is imperative. We report the prevalence of mutations in the *Plasmodium falciparum kelch 13* propeller domain associated with partial artemisinin resistance, which we determined by using Sanger sequencing samples from patients enrolled in therapeutic efficacy studies from 9 sub-Saharan countries during 2014–2018. Of the 2,865 samples successfully sequenced before treatment (day of enrollment) and on the day of treatment failure, 29 (1.0%) samples contained 11 unique nonsynonymous mutations and 83 (2.9%) samples contained 27 unique synonymous mutations. Two samples from Kenya contained the S522C mutation, which has been associated with delayed parasite clearance; however, no samples contained validated or candidate artemisinin-resistance mutations.

Malaria remains a serious global health concern, causing ≈405,000 deaths annually, mainly in young children in Africa (*1*). Although substantial progress has been made over the past decade to reduce the global burden of malaria, several factors threaten these gains, including the emergence and spread of antimalarial drug resistance (*1*). Artemisinin-based combination therapies (ACTs) are the first-line treatment for uncomplicated malaria caused by *Plasmodium falciparum* parasites, as recommended by the World Health Organization (WHO) (*2*). Unfortunately, resistance to ACTs (i.e., delayed parasite clearance and clinical treatment failures) has emerged in the Greater Mekong Subregion of Southeast Asia, posing a considerable risk to malaria control in the region (*3*). Even though clinical resistance to ACTs has not been reported in Africa (*1*), the threat of its emergence remains.

As part of antimalarial therapeutic efficacy activities, WHO recommends molecular surveillance of the *P. falciparum kelch 13* gene (*Pfk13*) (with focus on the propeller domain region), a molecular marker associated with delayed clearance of parasitemia after therapy with artemisinin monotherapy or an ACT (*3*–*7*). Because specific single-nucleotide polymorphisms (SNPs) within the propeller domain region of *Pfk13* continue to be discovered, WHO continues to update a list of these SNPs on the basis of association with delayed parasite clearance and reduced in vitro drug susceptibility (Table 1). Nine SNPs are currently considered validated by WHO to have delayed parasite clearance and in vitro data demonstrating partial resistance to artemisinin (*3*). WHO categorized 11 SNPs as candidate mutations, correlated with delayed parasite clearance but not validated with in vitro data (*3*). An additional 11 SNPs are listed by WHO as associated with delayed parasite clearance but without statistical significance because of limited data (*3*).

**Table 1:** Mutations in the *Pfk13* gene and WHO classification related to *Plasmodium falciparum* artemisinin resistance*

Validated *Pfk13* mutations	Candidate *Pfk13* mutations	Non–statistically significant associated *Pfk13* mutations
F446I	P441L	D452E
N458Y	G449A	C469Y
M476I	C469F	K479I
Y493H	A481V	R515K
R539T	P527H	S522C
I543T	N537I	N537D
P553L	G538V	R575K
R561H	V568G	M579I
C580Y	P574L, F673I, A675V	D584V, P667T, H719N

*Adapted from an August 2018 WHO status report on artemisinin resistance and artemisinin-based combination therapy efficacy (*3*). *Pfk13*, *P. falciparum kelch 13*; WHO, World Health Organization.

WHO recommends that malaria-endemic countries perform therapeutic efficacy studies (TESs) every 2 years to evaluate antimalarial treatments currently used in a particular region (*8*). Surveillance for molecular markers associated with antimalarial resistance is a recommended part of a TES to detect the presence of mutations associated with resistance (*8*). As part of the US President’s Malaria Initiative, the Centers for Disease Control and Prevention (CDC) and the US Agency for International Development provide support to countries in Africa to perform TESs, including molecular characterization of antimalarial-resistance markers, through the PMI-supported Antimalarial Resistance Monitoring in Africa (PARMA) network (*9*). Established in 2015, this endeavor involves laboratory trainees in Africa who bring TES samples from their home country to the CDC (Atlanta, Georgia, USA) to receive advanced laboratory training and perform molecular testing for antimalarial-resistance mutations (*9*). In this article, we report *Pfk13* mutation data generated from samples analyzed and collected from TESs conducted in 9 countries in Africa during 2014–2018.

## Methods

### Samples, Ethics Statement, and TES Protocols

Before initiation, all work described in this article was approved by the respective institutional ethics review committee in each country and the Office of the Associate Director of Science of CDC’s Center for Global Health and assigned the following tracking numbers: 2014–233a and 2014–233b (Angola), 2017–141 (Benin), 2018–035 (DRC), 2016–046 (Guinea), 6696.0 (Kenya), 6029.0 (Malawi), 2016–012a (Mali), 2015–073a (Tanzania), and 2016–200 (Zambia). Dried blood spots were collected from TESs conducted in 9 countries in Africa (Angola, Benin, the Democratic Republic of the Congo [DRC], Guinea, Kenya, Malawi, Mali, Tanzania, and Zambia; Table 2) during 2014–2018. The samples included those obtained pretreatment (at day of enrollment) and at day of treatment failure. Day of treatment failure samples came from patients experiencing a recrudescence or new infection during the follow-up period of (usually ending at 28 or 42 days) after administration of an ACT. TES and antimalarial molecular marker results for some of the data analyzed have been previously published for Angola (*10*–*12*), Kenya (*13*), and Tanzania (*14*). Results might differ slightly from previously published works because those works might not have reported results from all samples, might not have reported mutations in mixed infections, or might not have reported synonymous mutation results. Our study was a reanalysis of all available sequences using the same sequence data analysis quality filters, cut-offs, and quality scores for all countries.

**Table 2:** Summary of antimalarial therapeutic efficacy studies, 9 countries in Africa, 2002–2007*

Country	Sites	Treatments studied	Age of patients enrolled	Year	Total no. samples			ACTs introduced
					D0 + DF	D0	DF	
Angola	Benguela, Zaire, Lunda Sul	AL, ASAQ, DP	6 mo–12 y	2015	379	379	0	2005
				2017	76	38	38	2005
Benin	Klouanmey, Djougou	AL	6–59 mo	2017	194	175	19	2004
DRC	Kabondo, Kapolowe, Rutshuru, Mikalayi, Kimpese	AL, ASAQ, DP	6–59 mo	2017–2018	633	317	316	2006
Guinea	Maferinyah, Labè	AL	6–59 mo	2016	432	409	23	2004–2005
Kenya	Siaya County	AL, DP	6–59 mo	2016–2017	417	325	92	2006
Malawi	Machinga, Nkhotakota, Karonga	AL, ASAQ	6–59 mo	2014	27	8	19	2007
Mali	Dioro, Sèlinguè	AL, ASAQ	2–59 mo	2015–2016	410	320	90	2006
Tanzania	Kibaha, Ujiji, Mkuzi, Mlimba	AL	6 mo–10 y	2016	417	345	72	2006
Zambia	Gwembe, Katete, Mansa	AL, DP	>6 mo	2016	263	263	0	2002
Total					3,248	2,579	669	

*ACTs, artemisinin-based combination therapies; AL, artemether/lumefantrine; ASAQ, artesunate/amodiaquine; D0, day of enrollment (pretreatment); DF, day of failure; DP, dihydroartemisinin/piperaquine; DRC; Democratic Republic of the Congo.

### Sequencing of *Pfk13* Propeller Domain Region

We extracted DNA from dried blood spots using the QIAamp Blood DNA Kit (QIAGEN, https://www.qiagen.com) according to the manufacturer’s instructions. We amplified the propeller domain region from codon positions 389–649 by PCR and Sanger sequenced according to methods previously described (*15*).

### Data Analysis

We analyzed sequence data by using Geneious Prime (Biomatters, https://www.geneious.com). We trimmed and quality filtered forward and reverse sequence reads for each sample (error probability limit 0.05, maximum low-quality bases 30) from the 3′ and 5′ ends to remove low-quality bases. We aligned trimmed sequences to the *Pfk13* National Center for Biotechnology Information gene reference no. PF3D7_1343700 (https://www.ncbi.nlm.nih.gov/gene/814205) and assessed for SNPs. We only considered SNPs if they had a Phred quality score of >30 and were present in both forward and reverse strands. Mixed infections were detected by using the heterozygous caller plug-in tool in Geneious with a threshold of >30%. A second analyst confirmed all SNP and heterozygous calls by manual technical review. We submitted all *Pfk13* sequences with SNPs reported in this study to GenBank (accession nos. MN072940–3042). We used R software version 4.0.1 (R Foundation for Statistical Computing, https://www.r-project.org) to generate a map showing the distribution of mutations in the 9 countries (Figure).

**Figure:**
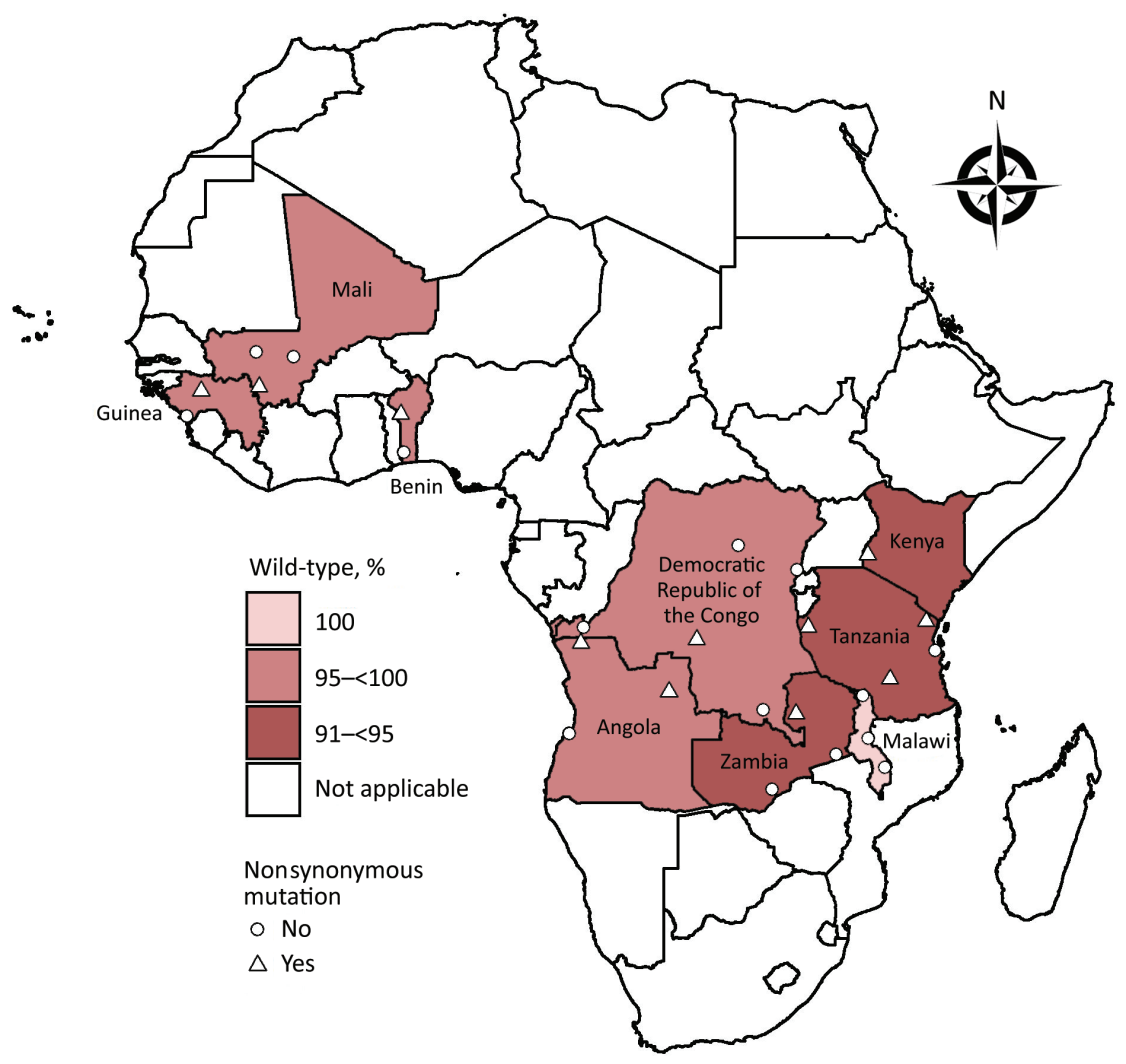
Prevalence of *Plasmodium falciparum kelch* *13* mutations in pretreatment therapeutic efficacy study samples, 9 countries in Africa, 2014–2018. A total of 11 unique nonsynonymous and 27 unique synonymous mutations were detected in 2,865 successfully sequenced pretreatment and day of failure samples from Angola, Benin, Democratic Republic of the Congo, Guinea, Kenya, Malawi, Mali, Tanzania, and Zambia collected during 2014*–*2018. A total of 2,753 samples were wild-type. Data from Angola includes results from 2 therapeutic efficacy studies.

## Results

We attempted *Pfk13* sequencing on 3,248 samples (2,579 pretreatment and 669 day of failure samples) from the 9 countries (Table 2); 2,865 were successfully sequenced (Table 3). Of those, 2,753 samples were wild-type. A total of 11 unique nonsynonymous mutations and 27 unique synonymous mutations were detected in 2,865 successfully sequenced pretreatment and day of failure samples from Angola, Benin, DRC, Guinea, Kenya, Malawi, Mali, Tanzania, and Zambia collected during 2014–2018 (Figure, Table 4; Appendix 1).

**Table 3:** Summary of *Pfk13* gene mutations detected in *Plasmodium falciparum* pretreatment and DF samples, 9 countries in Africa, 2014–2018*

	No. samples pretreatment (DF)					
Country (year)	Total with sequencing attempted	Poor quality or no amplification	Successfully sequenced	Wild-type samples pretreatment	Other nonsynonymous mutations	Synonymous mutations
Angola (2015)	379 (0)	77 (0)	302 (0)	291 (0)	5 (0)	6 (0)
Angola (2017)	38 (38)	0 (2)	38 (36)	37 (36)	1 (0)	0 (0)
Benin (2017)	175 (19)	20 (1)	155 (18)	151 (18)	1 (0)	3 (0)
DRC (2017–2018)	317 (316)	13 (34)	304 (282)	295 (269)	1 (2)	8 (11)
Guinea (2016)	409 (23)	20 (1)	389 (22)	380 (22)	1 (0)	8 (0)
Kenya (2016–2017)	325 (92)	7 (4)	318 (88)	302 (85)	8 (2)	8 (1)
Malawi (2014)	8 (19)	1 (5)	7 (14)	7 (14)	0 (0)	0 (0)
Mali (2015–2016)	320 (90)	68 (48)	252 (42)	244 (39)	1 (0)	7 (3)
Tanzania (2016)	345 (72)	20 (12)	325 (60)	306 (57)	6 (0)	13 (3)
Zambia (2016)	263 (0)	50 (0)	213 (0)	200 (0)	1 (0)	12 (0)
Total	2,579 (669)	276 (107)	2,303 (562)	2,213 (540)	25 (4)	65 (18)

*DF, day of failure; DRC, Democratic Republic of the Congo; *Pfk13*, *Plasmodium falciparum kelch 13*.

**Table 4:** Summary of *Pfk13* nonsynonymous mutations detected in *Plasmodium falciparum* pretreatment and DF samples, 9 countries in Africa, 2014–2018*

Mutation	Country	Codon change	No. samples pretreatment (DF)	Country or region where previously reported (reference)
I416V	Tanzania	ATA → GTA	1 (0)	Tanzania (*14*)
P419S	Guinea	CCA → TCA	1 (0)	NA
E433D	Tanzania	GAA → GAC	1 (0)	Tanzania (*14*)
R471S	Tanzania	CGT → AGT	1 (0)	Tanzania (*14*)
S477Y	DRC	TCT → TAT	0 (1)	Grande Comore Island (*16*)
A504V	Angola (2017)	GCT → GTT	1 (0)	Gabon (*17*)
S522C	Kenya	AGT → TGT	2 (0)	Africa (*18*)
A569G	Benin	GCA → GGA	1 (0)	Gambia (*19*) and Niger (*20*)
A578S	Angola (2015)	GCT → TCT	4 (0)	Africa (*19*)
A578S	DRC	GCT → TCT	1 (1)	Africa (*19*)
A578S	Mali	GCT → TCT	1 (0)	Africa (*19*)
A578S	Kenya	GCT → TCT	6 (2)	Africa (*19*)
A578S	Tanzania	GCT → TCT	1 (0)	Africa (*19*)
A578S	Zambia	GCT → TCT	1 (0)	Africa (*19*)
Q613R	Angola (2015)	CAA → CGA	1 (0)	NA
Q613E	Tanzania	CAA → GAA	2 (0)	Tanzania (*14*)
Total			25 (4)	

*DF, day of failure; DRC, Democratic Republic of the Congo; NA, not available; *Pfk13*, *Plasmodium falciparum kelch 13*.

Of the 2,303 sequenced pretreatment samples, 2,213 were wild-type and 90 (3.9%) contained mutations (Table 3). Of the 90 pretreatment samples with mutations, 10 unique nonsynonymous mutations were present in 25 samples from 8 of the 9 countries assessed (Table 4) and 25 unique synonymous mutations were present in 65 samples from 8 of the 9 countries assessed (Appendix 1 Table 1). Two samples from Kenya contained the S522C mutation, reported by WHO as a less-frequent mutation associated with delayed parasite clearance but without statistical significance because of limited data (*3*). Both of these patients cleared their initial infection. A578S, the most commonly found mutation in Africa (not associated with resistance) (*3*), was the most common nonsynonymous mutation we identified. The mutation was found in 14 pretreatment isolates: 4 in Angola, 1 in DRC, 1 in Mali, 6 in Kenya, 1 in Tanzania, and 1 in Zambia (Table 4). No mutations were identified in the samples from Malawi. Eight of the 10 unique nonsynonymous mutations in the pretreatment samples have been reported previously in other countries, whereas 2 mutations, P419S (Guinea) and Q613R (Angola), were newly identified in our study. No WHO-validated or candidate *Pfk13* mutations were identified.

Of the 669 day of failure samples, 562 were successfully sequenced; 107 (16.0%) samples failed to amplify, produced poor-quality sequences, or both (Table 3). A total of 540 samples were wild-type. Two nonsynonymous mutations were found in 4 day of failure samples (Table 4) and 10 synonymous mutations (Appendix 1 Table 2) were identified in 18 day of failure samples from 4 countries. Of the nonsynonymous mutations in day of failure samples, 2 samples from Kenya and 1 sample from DRC contained the A578S mutation, and 1 sample from DRC contained the S477Y mutation (Table 4). We compiled the complete results of the sequence data reanalysis (Appendix 2).

## Discussion

This work provides an update on *Pfk13* genetic markers in 9 countries in Africa with endemic malaria. Although clinical resistance to ACTs has yet to be confirmed in Africa (*1*), the early detection of *Pfk13* mutations through surveillance allows for swift action before resistance spreads widely. To date, all WHO-validated SNPs detected in Africa have been the result of independent emergence as opposed to spreading through imported cases from Southeast Asia (*21*). More than 200 *Pfk13* mutations have been identified in global samples (*3*,*18*,*21*), and >74 *Pfk13* nonsynonymous mutations have been reported in Africa (*22*,*23*). In this study, we report the presence of S522C in Kenya, a less frequent mutation that has been previously reported to be associated with delayed parasite clearance but lacking sufficient evidence to be considered a WHO-validated or candidate mutation (*3*).

As more molecular surveillance data are collected, previous results should be reinterpreted to determine the presence of WHO-reportable mutations because the importance of these mutations in drug resistance might change based on new data (*3*,*24*). Although we report only 1 mutation identified by WHO to possibly play a role in resistance, other detected mutations, such as the other nonsynonymous mutations with unknown resistance status reported in this study, might be deemed important in the future as more data are collected and validated. In 2017, WHO categorized only 5 mutations as validated (N458Y, Y493H, R539T, I543T, and 580Y) (*24*), but in 2018 the validated list was updated to include an additional 4 mutations, including F446I, P553L, and R561H (formerly candidate markers) and M476I (formerly reported as a less frequent variant associated with in vivo or in vitro test results) (*3*). In addition, the Worldwide Antimalarial Resistance Network tracks *Pfk13* mutations worldwide and strives to detect new associations of mutations with delayed parasite clearance, which might inform WHO classifications (*18*).

We report the presence of 11 unique nonsynonymous mutations in Angola, Benin, Guinea, DRC, Kenya, Mali, Tanzania, and Zambia; all were previously reported in the literature (Table 4) except P419S and Q613R. The most common nonsynonymous mutation observed in our study was A578S, a nonsynonymous mutation frequently described in Africa (*3*) and, to a lesser extent, Asia (e.g., Thailand [*19*] and Bangladesh [*25*]). WHO has reported that A578S is not associated with partial artemisinin resistance (*3*). Most mutations detected were synonymous mutations consistent with previous reports (*21*). Because synonymous mutations do not result in an amino acid change, they are not associated with resistance. Parasites from Africa have been shown to have a higher prevalence of synonymous mutations, which is not surprising given that *P. falciparum* originated in Africa and continues to have a high level of transmission in this region (*19*).

The results described in this article represent the collaborative output of the PARMA network, which originated in 2015 with the objectives of assisting countries in Africa in testing malaria samples from TESs for genetic markers associated with antimalarial resistance and supporting training and capacity building of collaborators in Africa (*9*). In 8 of the 9 countries included in this report (all but Angola), the *Pfk13* results were generated during a 6–8-week visit to CDC by trainees from a laboratory in the country where the TES was performed. Results were subsequently shared by the trainee’s laboratory with their national malaria control program and other local stakeholders to make decisions related to antimalarial use. Although the *Pfk13* results we have described would not be cause for alarm or policy change, recent findings in Rwanda suggests a substantial presence of the *Pfk13* R561H mutation (*26*) that has evolved locally, highlighting the importance of molecular surveillance for early detection of emerging patterns of resistance. In this context, PARMA training visits generate a vast amount of data from TES samples, ranging from efficacy results to prevalence of other molecular markers (e.g., *P. falciparum* multidrug-resistant protein 1 and *P. falciparum* chloroquine-resistance transporter) to the presence of *P. falciparum* histidine-rich protein 2 and 3 deletions (which might affect rapid diagnostic test performance). Generating phenotypic (i.e., efficacy) and genotypic data on the same sample provides an opportunity to identify novel mutations associated with resistance and enables detection of known mutations in samples with well-characterized efficacy outcomes. Because the PARMA network encourages standardization of laboratory methods and data reporting, such explorations might detect trends over time in a single country or produce insightful observations by using data from multiple countries. With the increased use of next-generation sequencing, the PARMA network has embarked on applying these principles of data generation, capacity building, networking, and standardization to this emerging technology (*27*). The ultimate goal is laboratories in Africa independently analyzing their own malaria samples.

Appendix 1 Additional information on *Plasmodium falciparum kelch* *13* mutations, 9 countries in Africa, 2014–2018.

Appendix 2 Reanalysis of sequence data and results in a study of *Plasmodium falciparum kelch 13* mutations, 9 countries in Africa, 2014–2018.
