# Supplementary material for: Plasmodium falciparum kelch 13 Mutations, 9 Countries in Africa, 2014–2018
Source: Emerg Infect Dis. 2021 Jul;27(7):1902–8. doi: 10.3201/eid2707.203230 (PMC8237877; doi:10.3201/eid2707.203230)
Supplement: Appendix 1 — Additional information on Plasmodium falciparum kelch 13 mutations, 9 countries in Africa, 2014–2018. [file 20-3230-Techapp-s1.pdf]

Article DOI: https://doi.org/10.3201/eid2707.203230

Plasmodium falciparum kelch 13 Mutations, 9 Countries in Africa, 2014–2018

Appendix 1

Appendix 1 Table 1. Summary of *Pfk13* synonymous mutations detected in pre-treatment samples from 9 countries in Africa*

Mutation	Country	Codon change	No. samples by country	No. samples total
P413P	Zambia, Mali	CCG → CCA	1, 1	2
P417P	Angola 2015, DRC, Kenya, Tanzania, Zambia	CCC → CCT	2, 2, 2, 3, 2	11
L429L	Guinea	TTG → CTG	1	1
V454V	Kenya	GTA → GTG	1	1
C469C	Angola 2015, Benin, Guinea, Kenya, Mali, Tanzania	TGC → TGT	1, 1, 3, 2, 2, 3	12
R471R	Angola 2015, DRC, Kenya, Tanzania, Zambia	CGT → CGC	2, 1, 1, 2, 3	9
S477S	DRC, Zambia	TCT → TCG	1, 2	3
T478T	Benin, Mali, DRC	ACC → ACG	1, 1, 1	3
V487V	Tanzania	GTA → GTG	4	4
Y493Y	Mali	TAC → TAT	1	1
G496G	Guinea	GGT → GGC	1	1
K503K	Mali	AAG → AAA	1	1
A504A	DRC, Kenya	GCT → GCC	1, 1	2
E509E	Guinea	GAG → GAA	1	1
V510V	Guinea	GTG → GTA	1	1
I526I	Angola 2015	ATA → ATT	1	1
R529R	Zambia	AGA → AGG	1	1
G533G	Kenya	GGT → GGA	1	1
R539R	Tanzania	AGA → CGA	1	1
A557A	Zambia	GCA → GCG	1	1
R575R	Zambia	AGA → AGG	2	2
S576S	DRC	TCA → TCG	2	2
V589V	Benin	GTC → GTA	1	1
Q613Q	Mali	CAA → CAG	1	1
A621A	Guinea	GCT → GCA	1	1
Total				65

*DRC, Democratic Republic of the Congo; *Pfk13*, *Plasmodium falciparum kelch 13*.

Appendix 1 Table 2. Summary of *Pfk13* synonymous mutations detected in day of failure samples from 4 countries in Africa*

Mutation	Country	Codon change	No. samples by country	No. samples total
P417P	DRC	CCC → CCT	1	1
C469C	DRC, Tanzania	TGC → TGT	1, 3	4
R471R	DRC	CGT → CGC	5	5
Y493Y	Mali	TAC → TAT	2†	2
G496G	DRC	GGT → GGC	1	1
Y511Y	DRC	TAT → TAC	1	1
G538G	Kenya	GGT → GGA	1	1
R539R	DRC	AGA → CGA	1	1
Q613Q	Mali	CAA → CAG	1	1
A621A	DRC	GCT → GCA	1	1
Total				18

*DRC, Democratic Republic of the Congo; *Pfk13*, *Plasmodium falciparum kelch 13*.
†Both day of failure samples were collected from the same patient at different time intervals post-treatment.
